# Random-start controlled ovarian stimulation for emergency fertility preservation in a patient with myelodysplastic syndrome: a case report

**DOI:** 10.1590/1414-431X20165227

**Published:** 2016-05-10

**Authors:** H. Cai, H. Shen

**Affiliations:** Reproductive Medical Center, Peking University People's Hospital, Beijing, China

**Keywords:** Emergency fertility preservation, Random-start, Ovarian stimulation, Myelodysplastic syndrome

## Abstract

This study reports a case of a gonadotropin-releasing hormone agonist trigger in a young female with myelodysplastic syndrome (MDS) who underwent fertility preservation using random-start controlled ovarian stimulation. This method involves the stimulation of the ovary regardless of a patient's menstrual-cycle phase. A review of the related literature is also provided. A 17-year-old patient was diagnosed with MDS and required initiation of peripheral blood stem cell transplantation within a maximum of 3 weeks and was in the luteal phase of the menstrual cycle when the possibility of attempting preservation of fertility was presented to her. She opted for a random-start controlled ovarian stimulation with gonadotropins. With successful hemorrhagic prophylaxis, 17 oocytes were retrieved including 10 mature and 7 immature oocytes. Of the immature oocytes, 3 were successfully matured *in vitro* and a vitrification protocol was used to freeze the 13 mature oocytes.

## Introduction

Myelodysplastic syndrome (MDS) includes a heterogeneous group of clonal hematopoietic stem cell disorders characterized by ineffective hematopoiesis and by bone marrow and peripheral blood morphological findings (1). Many cases of MDS have no symptoms and are diagnosed during a routine blood count test. The most prominent clinical features are anemia, neutropenia and thrombocytopenia (1). Patients with MDS can develop severe anemia and require blood transfusion. This disorder has a variable risk of transformation into acute leukemia and is considered a pre-leukemic state. Hematopoietic stem cell transplantation is thought to be a safe and effective option for patients with MDS. However, conditioning regimens always include high-doses of the antimetabolic agent cytarabine, which is harmful to the ovary. In a study on the incidence of and risk factors for fertility impairment, infertility was suspected in 83% of survivor women after hematopoietic stem cell transplantation in childhood and adolescence (2). Thus, the need is evident for an effective fertility preservation strategy that would allow these patients the possibility to conceive a child with their own gametes.

Currently, different strategies for fertility preservation in women, including embryo and oocyte cryopreservation, and ovary cryopreservation, are available. Ovarian tissue cryopreservation requires surgical procedures. Moreover, there is a risk for the malignant cell contamination of the graft, with reintroduction of the disease, particularly for hematological cancer diseases (3). Oocyte cryopreservation is indicated for postpubertal females that seek to delay pregnancy for a variety of reasons. In patients with MDS who need to start cytotoxic treatment soon, one of the biggest challenges in fertility preservation is the time required to complete the ovarian stimulation therapy. In these cases, protocols with alternative timing to start controlled ovarian stimulation (COS) have been proposed. Here, we report a case of a successful emergency oocyte cryopreservation in a young woman with MDS who was scheduled to undergo human leukocyte-associated antigen partially mismatched peripheral blood stem cell transplantation (PBSCT). As the PBSCT had to be initiated within a maximum of 3 weeks, the patient opted for random-start COS with gonadotropins during the luteal phase of the cycle with a satisfactory response.

## Case report

In 2009, a 17-year-old patient was referred to a local hospital with the complaint of persistent menorrhagia. Results of laboratory tests revealed thrombocytopenia (platelet count: 3.8×10^4^/µL). No family history of hematologic disorder was reported. Later she developed pancytopenia. Her bone marrow aspirate smear showed dysplastic features in trilineage cells without an increase in blast cells. Results of the cytogenetic study and the chromosome fragility test were negative. She needed repetitive blood transfusions. The oncologist performed leukocyte-associated antigen tests in the patient and her father, and PBSCT was planned and scheduled. After being informed and extensively counselled by a reproductive specialist with regard to oocyte cryopreservation, the patient decided to begin random-start COS for collection and preservation of gametes. This study was approved by the Ethics Committee of Peking University People's hospital. Informed consent was obtained from the patient and her parents.

The details of initial antral follicle count by ultrasound examination and hormonal assessment of the patient are reported in Table 1. In light of her imminent PBSCT, the patient did not have sufficient time to wait for the onset of the next menstrual cycle and the success of COS using conventional methods was unlikely. Alternatively, she immediately started treatment with recombinant follicle-stimulating hormone (FSH; Gonal-f; Sero, Germany) at a daily dosage of 225 IU. On day 20 of the menstrual cycle, dosages of FSH were adjusted based on estradiol (E_2_) levels and follicle size, to maximize follicular response. At approximately day 8 of gonadotropin stimulation, her follicles were consistently developed, with the lead follicles of about 12 mm, and on the same day, the serum luteinizing hormone (LH) level was 1.80 IU/L. Due to the absence of a premature LH surge, no gonadotropin-releasing hormone (GnRH) antagonist was administered. A single dose of 0.2 mg GnRH agonist (triptorelin; Ferring GmbH, Germany) was administered after 10 days of stimulation when the largest three follicles attained a mean diameter of 17 mm, with general cohort follicles >13 mm. The serum LH value was 0.71 IU/L on the day of ovulation. Transvaginal retrieval was performed 35 h after the administration of the GnRH agonist.

**Table:**
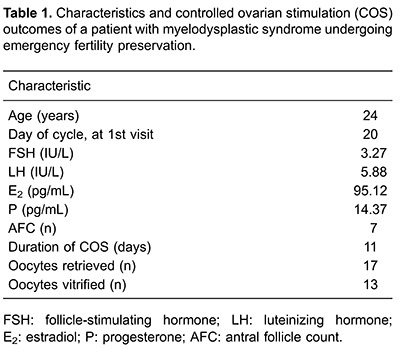

In the surgical room, the patient was sedated with pethidine hydrochloride (25 mg; Qinghai pharmaceutical Co., Ltd., China.) *im*, and perioperative antibiotics were administered (1.5 g cefuroxime sodium; Esseti Farmaceutici SRL, ltaly) immediately after sedation. A narrow ultrasound transducer (17G Oocyte Recovery Set; Smiths Medical International Ltd., UK) with an affixed needle guide was inserted vaginally, permitting transvaginal ovarian cyst puncture and aspiration. Seventeen oocytes were obtained. The patient was discharged after overnight observation without related complications. After 5 days, the patient had menstruation.

Of the 17 cumulus-oocyte complexes retrieved, 10 were mature and 7 immature. The immature oocytes were matured *in vitro* (IVM) and subsequently reassessed for maturity. Three of these oocytes attained nuclear maturity, and vitrification protocol was used to freeze the 13 mature oocytes.

## Discussion

In the present case report, 10 mature oocytes were retrieved after induction of ovulation, in concordance with the study by Courbiere et al. (4). Moreover, we were able to mature an additional 3 oocytes by IVM. This satisfactory response supports the effectiveness of emergency fertility preservation, in which oocytes can be obtained efficiently, irrespective of the phase of the menstrual cycle, in an urgent situation.

In a French multicenter cohort study, the leading indication for emergency *in vitro* fertilization was hematological cancer (42%) (4). However, limited data about fertility preservation choices and response to COS in patients with MDS are available in the literature (Table 2). Reichman et al. (5) described a successful ovarian stimulation and oocyte retrieval in a premenarcheal girl. A retrospective cohort study by Senapati et al. (6) reported 67 subjects with hematological disorders (5 had MDS). Tsai et al. (7) reported a live birth after single embryo transfer derived from autologous cryopreserved oocytes of a patient with MDS who had undergone allogenic PBSCT.

**Table:**
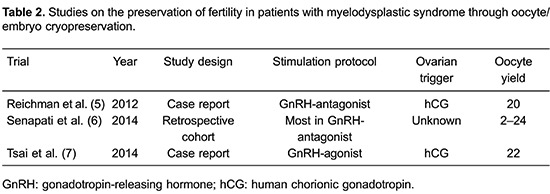

### Controlled ovarian stimulation (COS)

Conventionally, stimulation regimens in general infertility practice are started in the early follicular phase or after the pituitary blockade with a GnRH agonist. The ovarian stimulation for oocyte cryopreservation with GnRH antagonist is also initiated at the beginning of the follicular phase, which may require 2–6 weeks depending on the patient's menstrual cycle day.

### Random-start COS

In situations in which anti-cancer treatments must be initiated urgently, it is not desirable to wait for the next menstrual period to start a stimulation protocol; for such cases, random-start COS protocols have been proposed (8,9).

The following treatment plans are adopted depending on the phase of the menstrual cycles: If the patient is in the late follicular phase (menstrual cycle day 7 with emergence of a dominant follicle >13 mm, and/or progesterone level <2 ng/mL), ovarian stimulation with gonadotropins is started. When the secondary follicle cohort following stimulation reaches 12 mm, pituitary suppression with GnRH antagonist is initiated to prevent premature secondary LH surge and continued until the trigger (9). If the dominant follicle reaches 18 mm in diameter, ovulation is induced with hCG or GnRH agonist. After 2–3 days, the COS is started. If the patient is in the early luteal phase (progesterone level >3 ng/mL), ovarian stimulation is started without GnRH antagonist. The patient in the present study presented herself in this phase. In this young female, a decreasing trend in serum concentrations of LH was observed during the luteal phase. Hence, there was no need to administer the additional GnRH antagonist. However, the present protocol is different from the protocol suggested by Cakmak et al. (10). In their study, GnRH antagonist was administered to prevent premature secondary LH surge when the lead follicle reached 12 mm and was continued until the trigger. However, our clinical experience demonstrates that COS during the luteal phase resulted in lower serum LH concentrations on the day of the ovulation trigger; no patients presented a premature surge in LH. The suppression of LH secretion was likely the result of the increased value of progesterone (11). High concentrations of progesterone reduced the frequency of GnRH pulse, which further inhibited the secretion of LH and the occurrence of the LH surge, even though the circulating E_2_ concentration approached the threshold level at which an LH surge was generated by the positive feedback loop (11). The protocol used was in line with the recent observation by Kuang et al. (12) who provided evidence for the suppression of the luteal phase LH surge. This phenomenon simplifies ovarian stimulation protocols and makes it easier to monitor the procedure.

If the patient is in the mid-luteal phase, a GnRH antagonist is administered to induce regression of corpus luteum. After that, serum progesterone levels decrease and menses start 2–4 days later; hence, COS is started earlier instead of awaiting spontaneous menses (13).

Some researchers have evaluated the outcome of ovarian stimulation following conventional or random-start COS in patients with cancer. No differences were observed in the total dose of gonadotropins, numbers of oocyte retrieved, metaphase II oocytes when comparing the methods. The random-start approach was designed to allow the collection of oocyte in the shortest time possible, and is reported to be as effective as conventional COS (8,9).

## Conclusion

The present paper reports a case of a successfully performed random-start COS, which should be considered in patients who are not close to the first day of the menses and need an emergency fertility preservation. However, the efficacy of the strategy, especially in terms of future clinical pregnancy and live birth rates originating from the cryopreserved oocytes, awaits further research.
